# Behavioral state-dependent oscillatory activity in prefrontal cortex induced by chronic social defeat stress

**DOI:** 10.3389/fnins.2022.885432

**Published:** 2022-08-11

**Authors:** Tiaotiao Liu, Chengxi Qi, Wenwen Bai, Xin Tian, Xuyuan Zheng

**Affiliations:** School of Biomedical Engineering and Technology, Tianjin Medical University, Tianjin, China

**Keywords:** chronic social defeat stress, prefrontal cortex (PFC), behavioral state, local field potentials (LFPs), oscillatory activity

## Abstract

Chronic stress contributes to the onset and exacerbation of major depressive disorder (MDD) through the oscillatory activity in the prefrontal cortex (PFC). However, the oscillations on which chronic social stress converges to yield the behavioral state of social avoidance are largely unknown. Here, we use a chronic social defeat stress model and *in vivo* electrophysiological recordings to uncover a novel neurophysiological measure that predicts the social behavioral state in stressed animals. First, in this study, we find that chronic social defeat stress model induces depression-like behaviors (anhedonia and social avoidance). Second, we find statistically significant differences in PFC oscillatory activity across different frequency ranges in social behavioral state, and the oscillatory activity correlates with stress-induced behavioral state. Finally, we show that the social behavioral states are accurately decoded from the oscillatory activity based on machine learning. Together, these results demonstrate that naturally occurring differences in PFC oscillation underlie the social behavioral state that accompanies the emergence of stress-induced behavioral dysfunction.

## Introduction

Major depressive disorder (MDD) is a multifactorial mental disorder that is characterized by depressed mood, diminished interests, impaired cognitive function, and vegetative symptoms, such as disturbed sleep or appetite (Otte et al., 2016). It is known as the leading cause of disability in the world (Friedrich, 2017). Extensive literature suggests that stress contributes to the onset of MDD (Caspi et al., 2003; Woodward and Coutellier, 2021). Social stressors are known to control affective-like behavioral responses across a wide variety of mammalian species (Fuchs and Flugge, 2002). Repeated exposures to social defeat stress in rodents, for example, cause a robust depression-like phenotype marked by anhedonia, behavioral despair, and social-avoidance behaviors (Berton et al., 2006).

Chronic social defeat stress (CSDS) is a widely validated preclinical model of MDD (Krishnan et al., 2007; Chaudhury et al., 2013; Friedman et al., 2014). In this paradigm, test mice are repeatedly exposed to larger aggressive CD1 strain mice. At the end of these exposures, chronic social defeat stress induces a MDD-like behavioral syndrome characterized by anhedonia and social avoidance (Kudryavtseva et al., 1991; Berton et al., 2006).

Prefrontal cortex (PFC) has been recognized as an important region for stress response across several different research groups. Extensive literature has revealed the changes in PFC activity, plasticity, and gene expression following chronic stress exposure (Arnsten, 2015). Notably, the change in oscillatory activity in PFC has been linked with exposure to CSDS in recent study (Kumar et al., 2014). Nevertheless, the oscillations on which social stress converge to yield the behavioral state of social avoidance are largely unknown.

Therefore, in the present study, we recorded local field potential (LFP) in PFC in C57 mice before, and in response to, chronic social defeat stress. Then, neural oscillatory activity was quantified to reveal the manifestation of this neurophysiological profile in socially defeated mice. Then, the neurophysiological correlation between stress-induced changes in oscillatory activity and mice’s stress-related behavior was found. Finally, the characteristics of the oscillatory activity were involved in support vector classification to predict the mice’s behavioral states. Together, these results demonstrate that naturally occurring difference in PFC oscillation underlie social behavioral state that accompany the emergence of stress-induced behavioral dysfunction.

## Material and methods

### Animal care and use

C57BL/6J (C57) male mice and CD1 male mice were used throughout the study; they were purchased from SPF Biotechnology Co., Ltd. (Beijing, China, No. SCXK 2019-0010). The study was conducted using C57 mice that were 8–16 weeks old. All CD1 mice were retired male breeders, which were singly housed with environmental enrichment. All animals were maintained under a 12-h light-dark cycle in a climate-controlled environment (24°C, 50–55% humidity) with food and water available *ad libitum*.

All experimental procedures were conducted in accordance with the Guide for Care and Use of Laboratory Animals and were approved by the Tianjin Medical University Animal Care and Use Committee (license number: TMUaMEC2021060).

### Chronic social defeat stress

**FIGURE 1:**
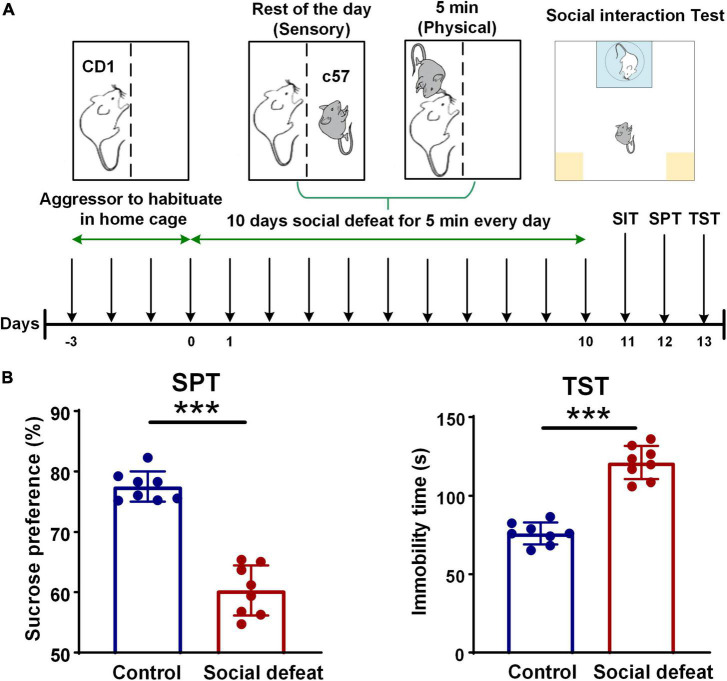
Schematic for CSDS paradigm and quality assessment of the social defeat model. **(A)** Experimental timeline showing all the steps of the experimental manipulations. **(B)** Quality assessment of the social defeat model (control, *n* = 8 mice; social defeat, *n* = 8 mice). In the SPT, the socially defeated mice showed a significant decrease in sucrose preference, compared with the control group (control: 77.5475 ± 0.8685; social defeat: 60.3250 ± 1.2877; unpaired t-test, *t* = 10.04, ***p < 0.001). In the TST, longer immobility time was found in the socially defeated mice, compared with controls (control: 76.0125 ± 1.9551; social defeat: 121.1125 ± 3.6877; unpaired t-test, *t* = 10.06, ***p < 0.001).

A schematic for the CSDS paradigm is shown in Figure 1A. Mice implanted with electrodes underwent 10 days of CSDS as previously described (Berton et al., 2006; Golden et al., 2011; Hultman et al., 2016). Specifically, male CD1 retired breeder mice were used as resident aggressors for the social defeat and were singly housed prior to the experiments. C57 mice were then randomly assigned to control or defeat groups such that no entire cage was assigned to the same group. All C57 mice were singly housed prior to being subjected to CSDS. Particularly aggressive CD1s, as defined by demonstrating at least one successful act of aggression toward an intruder C57 male within 60 s, were selected for use for CSDS. Intruder male C57 mice were introduced to the cage of a novel CD1 aggressor for 5 min daily and then housed adjacent to the same aggressor for 24 h. During this time, mice were separated by a transparent and porous Plexiglass barrier to enable constant sensory exposure (Kumar et al., 2014).

Following the last 24-h exposure to a CD1 aggressor mouse, all C57s were housed individually (Hultman et al., 2016). Control mice were pair-housed in the same cage with one mouse per side of the same transparent partition with perforated holes, but they did not experience physical contact with each other (Golden et al., 2011).

### Sucrose preference test

Sucrose preference test (SPT) were performed according to the published protocols (Krishnan et al., 2007; Liu M. Y. et al., 2018). Mice were first trained to consume 1% sucrose from two bottles for 24 h to acclimate them to sucrose, and then the animals were allowed free access to 1% sucrose and water from two bottles for 12 h. After 24-h deprivation of food and water, the animals were provided with 1% sucrose and water in two bottles. The mass of the fluid was weighed daily, and the positions of the bottles were interchanged to prevent possible effects of side preference in drinking behavior. The preference rate for sucrose (sucrose preference rate = sucrose consumption/total fluid consumption × 100%) was calculated and averaged over at least 2 days of testing.

### Tail suspension test

The Tail suspension test (TST) was used to analyze depressive behavior. Mice tails were individually suspended on a suspension shelf using a piece of adhesive tape. Each mouse was separated by wooden boards. The behavior of the mice was recorded for 5 min using a camera. Immobility, defined as complete motionlessness, was recorded.

### Social interaction test

Mice were placed within a novel arena (50 cm × 50 cm) with the “interaction zone” and the “corner zone.” The interaction zone of the test arena encompasses a 14 cm × 24 cm rectangular area projecting 8 cm around the wire-mesh enclosure. The corner zones encompass a 9 cm × 9 cm area projecting from both corner joints opposing the wire-mesh enclosure. Mice were first placed within the interaction zone, and each socially stressed mouse’s movement was monitored for 600 s. Mice were then removed from the testing chamber, and reintroduced 30 s later after a CD1 mouse was placed in the small cage. Locomotor activity measurements and time spent in the interaction zone and corner zone were quantified using the Ethovision XT 8.5 software (Noldus Information Technology, Wageningen, Netherlands). The social interaction ratio was calculated as (interaction time when CD1 was present)/(interaction time when CD1 was absent) (Golden et al., 2011; Kumar et al., 2014). Notably, only susceptible mice that showed social avoidance (the social interaction ratio < 1) were utilized for further oscillatory activity analysis.

### Electrode implantation surgery

Mice were anesthetized with 50 mg/kg IP of sodium pentobarbital, placed in a stereotaxic device, and metal ground screws were secured above the cerebellum and anterior cranium. A custom-made 16-channel microelectrode array (Liu T. et al., 2018; Tan et al., 2021) was implanted into the mPFC (prelimbic, PrL) of mice. The array was arranged in 4 × 4 configuration: 0.033 mm diameter nickel-chromium wires with formvar insulation (California Fine Wire Co., CA, United States), 0.25 mm interelectrode spacing, impedance <1 MΩ and gold-plated using IMP-2A (Bak Electronics Inc., FL, United States). Neurophysiological recordings were referenced to a ground wire connected to both ground screws. Histological analysis of implantation sites was performed at the end of experiments to confirm the recording sites used for neurophysiological analysis.

### Neurophysiological data acquisition

Neurophysiological recordings were performed using the Cerebus Acquisition System (Blackrock Microsystems Inc., UT, United States) during the social interaction test. Local field potentials (LFPs) were band-pass filtered at 0.5–250 Hz and stored at 2,000 Hz. All neurophysiological recordings were referenced to a ground wire connected to both ground screws.

### Local field potentials oscillatory power and correlation analysis

Power spectra were obtained by applying the short-time Fourier transform with a 0.5 s wide Hamming window and 0.5-Hz frequency smoothing to LFP signals. In order to test whether the power is related to the social avoidance behavioral in interaction/corner zone, we further analyzed the power (*Z*-score) in different frequency ranges relative to “No CD1” and “With CD1” phases. Moreover, Pearson’s correlation and linear regression analysis were used to calculate the relationship between the oscillatory activity and behavioral state.

### Classification using support vector machine

The support vector algorithm, a type of supervised machine learning algorithm, was used to classify the behavioral states based on the neuronal responses recorded in the different social behavioral states. The behavioral states can be divided into four categories, including normal in the interaction zone, normal in the corner zone, socially defeated in the interaction zone, and socially defeated in the corner zone). Here, we used support vector classification with a quadratic kernel for all decoders (Grundemann et al., 2019). The predictions were made depending on the 10 measures of oscillatory activity in 5 different frequency ranges from PFC. The dataset includes data from 396 total epochs under four behavioral states, recorded from the socially defeated mice (*n* = 8) and control (*n* = 8). For testing the accuracy of the classification, the dataset was randomly divided into training (80%) and test (20%) sets. Since the accuracy is a metric used in classification problems, it is used to tell the percentage of accurate predictions. To measure the performance of our SVM model, we calculated the accuracy by dividing the number of correct predictions by the total number of predictions.

### Statistical analysis

All the data are represented as the mean ± SEM. An unpaired t-test was used to analysis the behavioral results between control and social defeat group. The statistical difference of the power changes was calculated by two-way ANOVA, followed by *post hoc* comparisons with Bonferroni’s test. Pearson’s correlation and linear regression analysis were used to evaluate correlations. Statistical tests and test statistics are mentioned in the text and figure legends. *, **, and *** indicate *p* < 0.05, *p* < 0.01, and *p* < 0.001, respectively.

## Results

### Chronic social defeat stress model induces depression-like behaviors and social avoidance

We exposed 14 male C57BL/6J mice to 10 days of CSDS to mimic depression and observed the CDS induced a depression-like phenotype (anhedonia) in 8 mice. To examine the effect of CSDS on depression-related behavior, SPT and TST were conducted after the social defeat procedures. In the SPT, sucrose preference was decreased in socially defeated mice (Figure 1B, left). After CSDS procedures, the socially defeated mice showed a significant decrease in sucrose preference, compared with the control group. Additionally, in the TST, the socially defeated mice had a significantly longer immobility time compared with controls, indicating aggravated depressive-like behavior (Figure 1B, right). These results of the behavioral tests indicated that the socially defeated mice developed a depression phenotype after CSDS.

**FIGURE 2:**
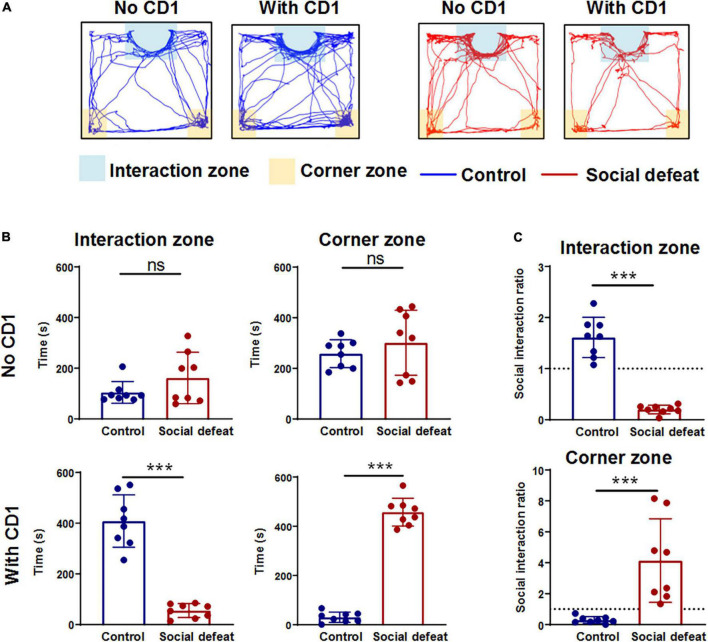
Effects of chronic social defeat stress on mice social behaviors. **(A)** Representative activity tracking during the social interaction test for control and socially defeated mice in the absence or presence of a CD1 target. **(B)** Summary plots of time in interaction zone and time in corner zone of the two groups in the absence or presence of a CD1 target (control, *n* = 8 mice; social defeat, *n* = 8 mice). **(C)** Plots of the social interaction ratio in interaction zone and corner zone for control and socially defeated mice. All data are presented as the mean ± SEM. ***p < 0.001. Time in interaction zone, No CD1: (control: 258.2414 ± 18.2320; social defeat: 301.3191 ± 42.5101; unpaired *t*-test, *t* = 0.8712, *p* = 0.3984). Time in corner zone, No CD1: (control: 104.6443 ± 14.1879; social defeat: 161.5693 ± 33.5602; unpaired *t*-test, *t* = 1.4610, *p* = 0.1660). Time in interaction zone, With CD1: (control: 408.3468 ± 34.1674; social defeat: 55.2361 ± 9.0972; unpaired *t*-test, *t* = 9.3420, ***p < 0.001). Time in corner zone, With CD1: (control: 30.4027 ± 6.9439; social defeat: 457.2912 ± 18.7106; unpaired *t*-test, *t* = 20.010, ***p < 0.001). Social interaction ratio in interaction zone: (control: 1.6102 ± 0.1300; social defeat: 0.2004 ± 0.0276; unpaired *t*-test, *t* = 9.919, ***p < 0.001). Social interaction ratio in corner zone: (control: 0.2966 ± 0.0722; social defeat: 4.1410 ± 0.8949; unpaired *t*-test, *t* = 4.0060, ***p < 0.001).

After 10 consecutive days of defeat stress, the social interaction test was used to explore the effects of chronic social defeat stress on mice social behaviors (Figure 2A). Compared with the control mice, the socially defeated mice spent less time in the interaction zone and more time in the corner zone when the CD1 mouse was present in the mesh enclosure (Figure 2B). Furthermore, the time spent in the interaction zone and corner zone without a CD1 mouse showed no significant difference (Figure 2B). Social avoidance behavior can also be expressed as a social interaction ratio. The social interaction ratio in the interaction zone of the socially defeated mice was significantly lower than that of the control, while the social interaction ratio in the corner zone of the socially defeated mice was significantly higher than that of the control (Figure 2C). These results suggest that chronic social defeat stress leads to social avoidance behavior (see also in Supplementary Figure 1).

### Effects of social defeat on oscillatory activity in prefrontal cortex

**FIGURE 3:**
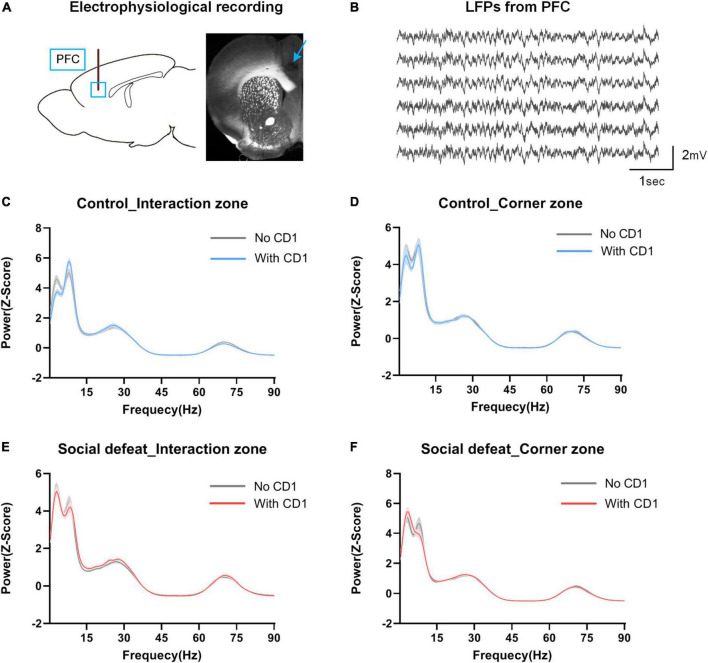
Power distribution in PFC in the social interaction test. **(A)** Schematic of electrophysiological recording site. Blue box and arrow represent the multichannel electrodes recording site (prelimbic cortex). **(B)** Sample LFP traces recorded from PFC. **(C,D)** Spectral power in the interaction zone and corner zone for controls. **(E,F)** Spectral power in the interaction zone and corner zone for the socially defeated mice. Data are shown as mean ± SEM. Note the oscillatory signals within the 2–12 Hz range were highly redundant in both “With CD1” and “No CD1” phases in the interaction zone and corner zone, and the distribution shows a bimodal distribution.

Neurophysiological activity was recorded from multielectrodes implanted in the PFC during the social interaction test (see Figure 3A for implantation sites; Figure 3B, for example, LFP traces). To evaluate the neurophysiological responses to CSDS, we first analyzed the PFC oscillatory activity in the different behavioral states during the SIT.

As can be seen from Figures 3C–F, high spectral power was observed within the 2–12 Hz frequency band during all periods of the social interaction test (in the interaction zone and corner zone, with and without the introduction of the aggressor CD1 mouse). The 2-12 Hz power distribution showed an obvious “bimodal” distribution, which can be roughly divided into 2–7 Hz and 7–12 Hz.

**FIGURE 4:**
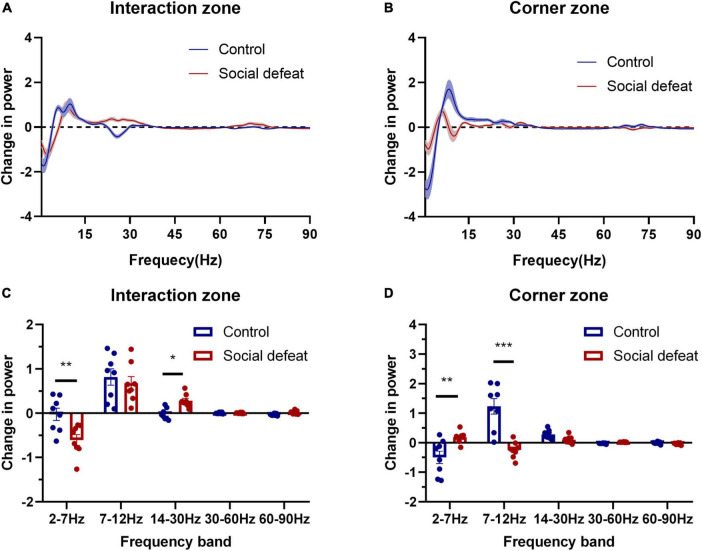
Oscillations in different frequency ranges are associated with social avoidance. **(A)** Change in power in the interaction zone. **(B)** Change in power in the corner zone. **(C)** The two groups showed statistical differences in the interaction zone (*F*(1,7) = 5.483, *p < 0.05, two-way ANOVA, *post hoc* comparisons with Bonferroni’s test). Note the 2–7 Hz oscillation of the socially defeated mice showed a negative change while 14–30 Hz oscillation showed a positive growth, compared with controls (*p < 0.05, **p < 0.01). **(D)** The two groups exhibited statistical differences in the corner zone (*F*(1,7) = 6.088, *p < 0.05, two-way ANOVA, *post hoc* comparisons with Bonferroni’s test). Note the 2–7 Hz and 7–12 Hz oscillations of the socially defeated mice experienced relatively minor changes (**p < 0.01, ***p < 0.001).

We further made a statistical comparison in spectral power (*Z*-score) across different frequency ranges (Figure 4). Here, evaluated changes in oscillatory power was quantified as the difference in each power with and without the CD1, which was introduced during the social interaction test (Change in power = Power_With CD1_ – Power_No CD1_).

The statistical results revealed that the 2–7 Hz oscillation of the socially defeated mice showed a negative change while 14–30 Hz oscillation showed a positive change, compared with controls in the interaction zone. Moreover, the 2–7 Hz and 7–12 Hz oscillations of the socially defeated mice experienced relatively minor changes. These results suggest that PFC oscillatory activity was correlated with the social behavioral state changes induced by CSDS.

### Prefrontal cortex oscillatory activity correlates with stress-induced behavioral state

**FIGURE 5:**
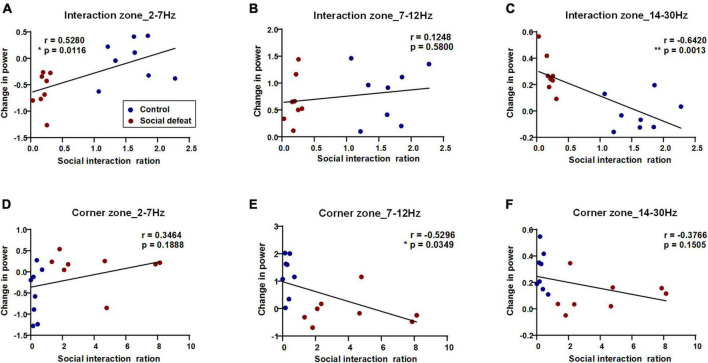
PFC oscillatory activity in the social interaction test predicts the social interaction ratio. Pearson’s correlations and linear regression between power changes in different frequency ranges and behavioral states were shown (blue dots: control, *n* = 8 mice; red dots: social defeat, *n* = 8 mice). **(A)** Interaction zone, 2–7 Hz, correlation value: 0.5280, *p = 0.0116; **(B)** Interaction zone, 7–12 Hz, correlation value: 0.1248, *p* = 0.5800; **(C)** Interaction zone, 14–30 Hz, negative correlation value: – 0.6420, **p = 0.0013; **(D)** Corner zone, 2–7 Hz, correlation value: 0.3464, *p* = 0.1888; **(E)** Corner zone, 7–12 Hz, negative correlation value: – 0.5296, *p = 0.0349; **(F)** Corner zone, 14–30 Hz, negative correlation value: – 0.3766, *p* = 0.1505.

To characterize the relationship of PFC oscillatory activity to the behavioral states, we set out to conduct Pearson’s correlation analysis and linear regression analysis to investigate the correlation between the power changes in different frequency ranges and the social interaction ratio (Figure 5). Specifically, in the interaction zone, the power change in 2–7 Hz was positively correlated with the social interaction ratio (*p < 0.05), while the power change in 14–30 Hz was negatively correlated with the social interaction ratio (**p < 0.01). In the corner zone, the power change in 7–12 Hz was negatively correlated with the social interaction ratio (*p < 0.05).

### Behavioral state decoding using machine learning

**FIGURE 6:**
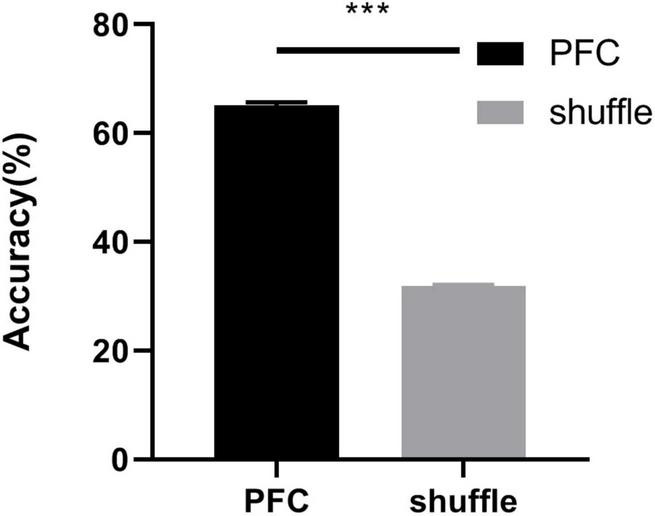
Decoder accuracy for real PFC data and shuffled data. The classification accuracy was evaluated using a support vector machine jointly discriminated the social behavioral states (real data: 65.16% ± 0.51%, shuffled data: 31.87% ± 0.20%, unpaired *t*-test, ***p < 0.001).

To test whether the social behavioral states could be accurately decoded from the oscillatory activity of PFC, we trained a decoder based on support vector machine on the distinction between social interaction zone and corner zone, with and without CD1. The observed LFP patterns include spectral power across frequencies and were involved in the machine learning algorithm. To control for the local dependencies in both the behavioral data and LFPs, we repeated the decoding training procedure with the behavior circle-shifted relative to the neuronal activity for 1,000 random shifts (Grundemann et al., 2019). As shown in Figure 6 The decoder performance was high when training and testing were performed on the real data (decoder accuracy = 65.16% ± 0.51%) and dropped when training and testing were performed on shuffled data (decoder accuracy = 31.87% ± 0.20%). The results indicated that the LFP patterns of the socially defeated mice in different social behavioral states had obvious distinguishing characteristics and could be decoded by the power changes of LFPs in different frequency ranges.

## Discussion

In this study, using a chronic social stress model and *in vivo* electrophysiological recordings, we characterized the changes in PFC oscillatory activity in response to exposure to an aggressor mouse and correlated them with social behavioral differences in chronically socially defeated mice. Our results revealed the behavioral state-dependent oscillatory activity in the PFC after exposure to chronic social defeat stress.

The behavioral tests provide direct evidence that the socially defeated mice developed a depression-like phenotype (anhedonia and social avoidance) after CSDS procedures. Meanwhile, the statistical comparisons in spectral power across different frequency ranges showed the differences in PFC oscillatory activity in the stressed mice. These results are consistent with the previous studies on social stress-induced behavioral deficits and altered neural activity in the PFC. Regarding the underlying mechanism, research scientists have done a lot of work to uncover its nature of the structure and function of molecular biochemistry and other perspectives. Specifically, CSDS induces sustained dendritic and synaptic structural changes in the PFC (McEwen et al., 2015; Colyn et al., 2019), which are significantly important for maintaining the oscillatory activity. Moreover, the functional change of PFC myelination is also regarded as a critical determinant of the avoidance response to traumatic social experiences (Bonnefil et al., 2019). Advancement in molecular research revealed that social defeat stress specifically increases c-Fos expression (a marker for neuronal activity) in the PFC (Numa et al., 2019), and social stress-induced behavioral deficits are mediated by molecular adaptations in the prefrontal cortical circuit involving ΔFosB and cholecystokinin (Vialou et al., 2014). Besides, dopamine D2 receptor dimerization (Bagalkot et al., 2015) and astrocytic glycogen accumulation (Zhu et al., 2021) in PFC is also closely related to stress-induced depression-like behavior.

In this study, we further divided the behavioral states into interaction zone and corner zone to study the changes in oscillatory activity during social exploration. An interesting result is the distinguished differences in oscillation across frequency ranges. Specifically, in the interaction zone, the power change in 2–7 Hz was positively correlated with the social interaction ratio, while the power change in 14–30 Hz was negatively correlated with the social interaction ratio. Our results show a significant difference in 2–7 Hz power in the interaction zone and corner zone in socially defeated mice during exposure to an aggressor mouse. Specifically, the 2–7 Hz power increases in the corner zone while decrease in the interaction zone. It should be noted that the socially defeated mice spend much more time in the corner zone. When taking the time factor into account, the total change in power can be calculated. The total change in power is positive, indicating an increase in 2–7 Hz power in susceptible mice during exposure to an aggressor CD1 mouse, which was consistent with previous reports (Kumar et al., 2014). Long-term stress exposure leads to architectural changes in the PFC and may alter its functional connectivity to the rest of the brain. Importantly, PFC activity preceded other regions at 2–7 Hz. Moreover, PFC and AMY (amygdala) exhibited directionality in 14–23 Hz (Hultman et al., 2016), and the PFC–AMY circuit is recognized to reflect the activation of a feedback regulatory network that suppresses subcortical neurophysiological responses to stress.

To test whether the social behavioral states could be accurately decoded from the oscillatory activity of the PFC, we trained a decoder based on a support vector machine on the distinction between the social interaction zone and corner zone, with and without an aggressor. The observed LFP patterns include spectral power across 5 frequency bands and were involved in the machine learning algorithm. Compared with shuffle, the decoder performance was high (accuracy = 65.16%) when training and testing were performed on the LFPs. The results indicated that the oscillatory patterns in different social behavioral states were remarkably distinct and could be decoded by the oscillatory activity. Thus, the changes in oscillatory activity can be used as a neurophysiological marker, which can be assessed at the level of LFPs, allowing for reliable and rapid classification of animal behavioral states in the social interaction test. However, the accuracy of the decoder still needs to be further improved. Though the regulation of emotional behavior is highly dependent on PFC, PFC also makes direct monosynaptic connections with multiple limbic brain regions, including AMY and VTA (ventral tegmental area; Oh et al., 2014), and these PFC-dependent circuits have been revealed to regulate the emotional behavior. Furthermore, we aimed to study the oscillatory activity and directionality among multiple brain regions, which could be involved in decoding the stress-induced behavioral state more accurately.

Our findings demonstrate that, in mice, the PFC neural oscillation correlates with the behavioral responses that occur in response to chronic social defeat stress. Importantly, we described the changes in oscillatory patterns as a novel neurophysiological marker that can be used to classify the social behavioral states. Thus, the use of such a neurophysiological biomarker enables a deeper investigation into the molecular- and cellular-based brain mechanisms that ultimately determine individual behavioral responses to stress.

## Data availability statement

The raw data supporting the conclusions of this article will be made available by the authors, without undue reservation.

## Ethics statement

The animal study was reviewed and approved by Tianjin Medical University Animal Care and Use Committee (License number: TMUaMEC2021060).

## Author contributions

TL, XZ, and XT designed the experiments. TL and CQ performed the experiments. TL analyzed the data. TL and WB wrote the manuscript. All authors participated in revising it and approved the final version.
