# Supplementary figures and images for: Behavioral state-dependent oscillatory activity in prefrontal cortex induced by chronic social defeat stress
Source: Front Neurosci. 2022 Aug 11;16:885432. doi: 10.3389/fnins.2022.885432 (PMC9403768; doi:10.3389/fnins.2022.885432)

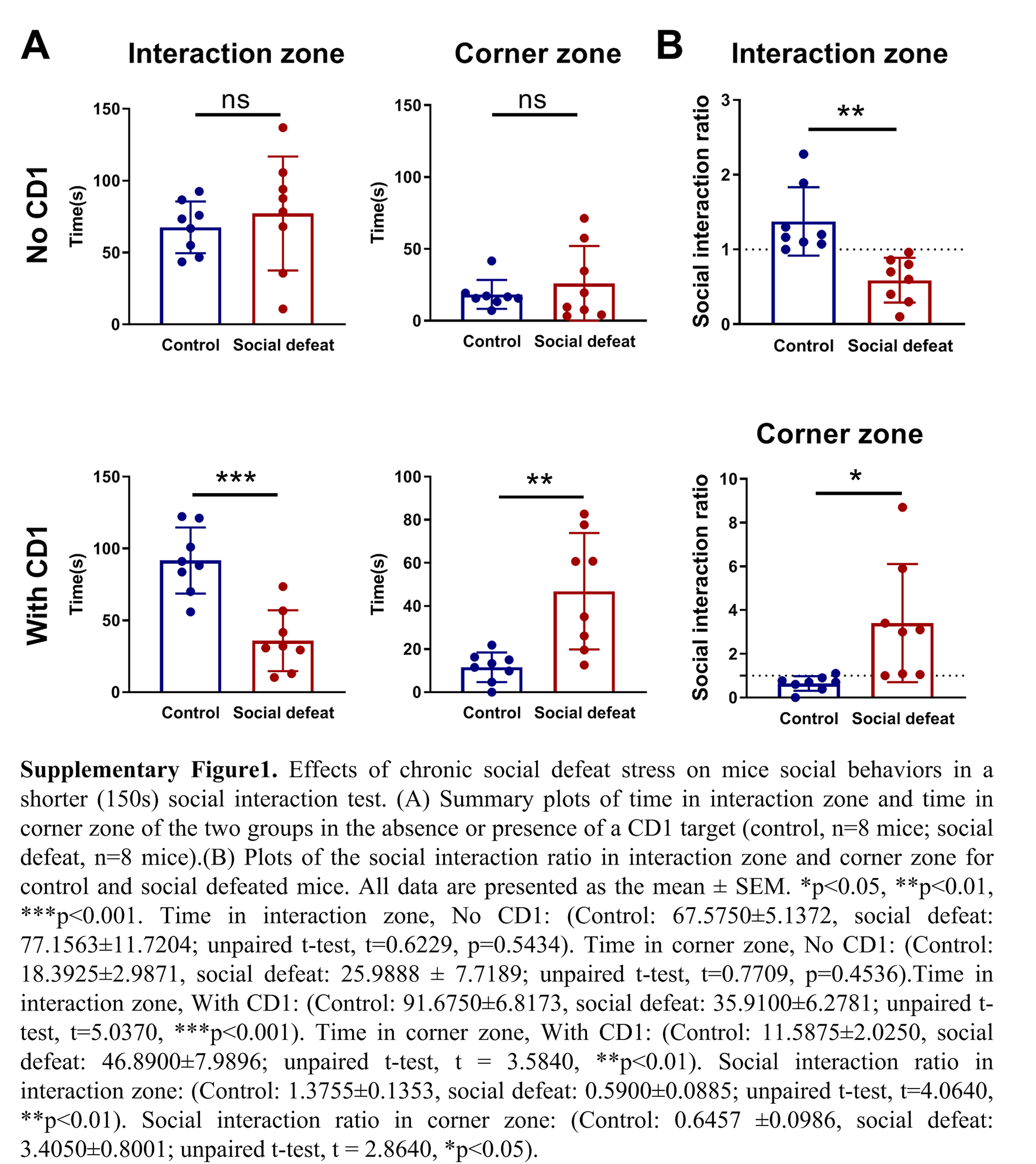

Supplement: Supplementary file 1 [file Image_1.TIF]
